# Isolation and characterization of hybrid poplar galactinol synthases

**DOI:** 10.1186/1753-6561-5-S7-P77

**Published:** 2011-09-13

**Authors:** Faride Unda, Shawn Mansfield

**Affiliations:** 1 Wood Science Department -University of British Columbia, Canada; 2 Wood Science Department- University of British Columbia, Canada

## 

The raffinose family of oligosaccharides (RFOs) likely fulfill at least few major physiological roles in plants, translocation of carbon in the phloem, storage in sink tissues, and as putative biological agents to combat both abiotic and biotic stress [1-3]. The synthesis of galactinol from *myo*-inositol + UDP-galactose, by galactinol synthase (GolS), is considered a key regulatory step in RFO synthesis [4].To investigate the functional roles of this class of compounds in trees, two cDNAs that encode galactinol synthase (GolS), were identified and cloned from hybrid poplar (*P. alba × grandidentata*). Phylogenetic analyses of the *Populus* GolS isoforms with other known galactinol synthases suggested a putative role for these enzymes during biotic or abiotic stress in hybrid poplar. The predicted protein sequences of both isoforms (PaxgGolSI and PaxgGolSII) showed characteristics of galactinol synthases from other species, including a serine phosphorylation site at position 266 and the pentapeptide hydrophobic domain ASAAP [5]. Kinetic analyses of recombinant Pa×gGolSI and PaxgGolSII resulted in K_m_ values for UPD-galactose of 0.79 and 0.65 mM and V_max_ values of 660.4 and 1245 nM min^-1^, respectively. PaxgGolSI inherently possessed broader pH range and temperature sensitivity when compared to Pa×gGolSII. Interestingly, spatial and temporal expression analyses revealed that *PaxgGolSII* transcript levels varied seasonally, while *PaxgGolSI* did not, thereby implying a temperature-regulated transcriptional control of this gene in addition to the observed thermosensitivity of the respective enzyme. Based on this evidence, we suggest that Pa×gGolSI may be involved in basic metabolic activities (*i.e.*storage), while Pa×gGolSII is likely involved in seasonal mobilization of carbohydrates.
